# Research note: Marek's disease oncogene *Meq* directly regulates *CD30* high expression through binding to two regions in promoter

**DOI:** 10.1016/j.psj.2025.106360

**Published:** 2025-12-28

**Authors:** Yushuang Du, Gang Zheng, Yuqin Yang, Jiannan Zhang, Juan Li, Ling Lian

**Affiliations:** a Frontiers Science Center for Molecular Design Breeding, China Agricultural University, Beijing 100193, China; b State Key Laboratory of Farm Animal Biotech Breeding, China Agricultural University, Beijing 100193, China; c Key Laboratory of Bio-Resources and Eco-Environment of Ministry of Education, College of Life Sciences, Sichuan University, Chengdu 610065, China

**Keywords:** Chicken, Marek's disease, *CD30*, *Meq*, Transcriptional regulation

## Abstract

Marek's disease (MD), caused by the oncogenic Marek's disease virus (MDV), is a lymphoproliferative disease in chickens and serves as a natural animal model for *CD30*-overexpressing lymphomas. The expression of *CD30*, a member of the tumor necrosis factor receptor superfamily, is significantly upregulated during lymphocyte transformation in MD. The MDV-encoded proto-oncogene *Meq* is known to bind to the chicken *CD30* promoter, but the precise regulatory mechanisms and functional binding sites remain uncharacterized. This study aimed to elucidate the transcriptional regulation of *CD30* by *Meq.* Initially, expression of *CD30* was detected in spleens of MDV-uninfected chickens and tumorous spleens of MDV-infected chickens, and the results showed it was highly expressed in tumorous spleens of MDV-infected chickens. Knockdown of *Meq in vitro* resulted in a corresponding downregulation of *CD30* expression, confirming a positive regulatory relationship between them. Subsequently, dual-luciferase reporter assays and chromatin immunoprecipitation (ChIP)-qPCR demonstrated that *Meq* directly bound to the *CD30* promoter to activate its transcription, and the primary binding regions located at -1383 to -1162 bp and -97 to +75 bp relative to the transcription start site. Collectively, this study demonstrated *Meq* could bind to *CD30* promoter at two binding regions and up-regulate its expression, and provided a critical foundation to investigate the role of the *CD30* signaling pathway in MD-induced tumorigenesis.

## Introduction

Marek’s disease (MD) is a highly contagious, oncogenic disease of poultry caused by Marek’s disease virus (MDV), a member of the Herpesviridae family. MDV is classified as a lymphotropic oncogenic herpesvirus, capable of inducing lymphoid cell proliferation and transformation, ultimately leading to the development of T-cell lymphomas in chickens. The lymphoma in MD offers a unique *in vivo* model to investigate virus–host interactions and the molecular pathways of malignant transformation, thereby providing valuable insights into oncogenic signaling (Biggs and Nair, 2012). Notably, elevated *CD30* expression is a hallmark of lymphocyte transformation into malignant cells, a phenomenon conserved across species and associated with multiple lymphoma subtypes. This observation suggests that *CD30* is involved in driving lymphocyte tumorigenesis (Burgess et al., 2004). Given its consistent association with lymphocyte malignancies, *CD30* emerges as a key molecule warranting further investigation to elucidate its role in MD and related oncogenic processes.

*CD30* is also known as Tumor Necrosis Factor Receptor Superfamily Member 8 (*TNFRSF8*), encodes a transmembrane glycoprotein belonging to the tumor necrosis factor receptor superfamily. *CD30* plays a pivotal role in immune regulation and cellular signal transduction, influencing processes such as lymphocyte activation, proliferation, survival, and apoptosis. It is predominantly expressed on activated T cells and B cells, and upon binding to its ligand *CD30L*, activates downstream signaling cascades-including *NF-κB* and *MAPK* pathways—that modulate cellular fate (Nakashima and Uchimaru, 2023). The functional studies using *CD30*-deficient mice have revealed that while *CD30* is dispensable for lymphocyte development, and it plays an essential role in T cell-dependent immune responses and in shaping the tumor microenvironment (Cui et al., 2024). In avian species, *CD30* is markedly upregulated in transformed T lymphocytes, making MDV-induced lymphoma a natural *CD30*-high tumor model. This provides a unique opportunity to investigate *CD30* role in virus-induced lymphomagenesis within a natural host system. Although *CD30* is consistently implicated in lymphocyte malignancies, its precise functional mechanisms in the context of MD remain incompletely understood, warranting further exploration of the molecular pathways underlying its regulation.

*Meq* is an important oncogenic determinant. Emerging evidence indicates that *Meq* expression correlates positively with *CD30* expression in MDV-induced lymphomas, and CD30-specific Ig is detected after MDV infection of MD-resistant chickens (Burgess et al., 2004). Although prior studies have predicted *Meq* binding with the regulatory regions of the chicken *CD30* gene, the precise binding sites and transcriptional regulatory mechanisms remain undefined. In this study, we investigated the regulatory relationship between *Meq* and *CD30* through *in vitro* experiments, confirming that *Meq* functions as a transcription factor capable of modulating *CD30* expression.

## Material and methods

### Cell culture

The DF-1 cells and HEK293T cells used in this experiment were purchased from Shanghai Jinyuan Biotechnology Co., Ltd. (Shanghai, China). The cells were cultured in Dulbecco’s modified Eagle’s medium (DMEM, Gibco, USA) supplemented with 5 % fetal bovine serum (FBS, Gibco, USA) and 1 % penicillin/streptomycin (PS, Gibco, USA). MDV-transformed lymphoid cell line, MSB-1, was kindly provided by Dr. C. Itakura, and has been maintained in our laboratory, cultured in RPMI-1640 medium (Gibco, USA) supplemented with 10 % FBS and 1 % PS.

### Tissue samples

Spleen tissues used in the present study were from our previous work (Lian et al., 2010), including the spleens from specific-pathogen-free (SPF) chickens (*N* = 20) experimentally infected with MDV-GA strain (2,000 plaque-forming units), and spleens from non-infected SPF chickens (*N* = 20).

### Primer design

Based on the chicken *CD30* (GenBank ID: NC_052552.1) and *Meq* (GenBank ID: NC_002229.3) sequences available on NCBI (https://www.ncbi.nlm.nih.gov/), RT-qPCR primers for *CD30* and *Meq*, PCR amplification primers for the *CD30* promoter truncation assay, and primers for the *CD30* immunoprecipitated assay were designed using Primer Premier 5. Primers were synthesized by BGI (Beijing, China). The specific primer sequences were shown in Table 1.

**Table 1:** Primer sequences for RT-qPCR, vector construction, and RNA oligo sequences.

Primers	Sequences (5′−3′)
*CD30*-QP^1^-F	CCTTTCAAGTGCCACAACCG
*CD30*-QP-R	GGCGTTTTTGAAGACCCCAC
*Meq*-QP-F	GGAGCCGGAGAGGCTTTATG
*Meq*-QP-R	ATCTGGCCCGAATACAAGGAA
*CD30*-PRO^2^-F	CTGGCCTCGGCGGCCAAGCTTTAGTACTTCTCCTCGGTGTATTGTGGTGC
*CD30*-PRO-R	CAGTACCGGATTGCCAAGCTTCTGCAGTGGCGTTAGCTTTCCTGATCTCCCA
*CD30*-PRO-F1	CTGGCCTCGGCGGCCAAGCTTAGCAACTTTATCCTCACCATTTCT
*CD30*-PRO-F2	CTGGCCTCGGCGGCCAAGCTTTGATATTTGGGCATTCGAATGCTTA
*CD30*-PRO-F3	CTGGCCTCGGCGGCCAAGCTTGTTTAAACGGCAACGTGCAGGA
*Meq*-CDS^3^-F	CTAGCGTTTAAACTTAAGCTTATGTCTCAGGAGCCAGAGCC
*Meq*-CDS-R	TCCGAGCTCGGTACCAAGCTTTCAGGGTCTCCCGTCACCTG
IP^4^-F1	GGCTTTTCCGTTTGTCTGGC
IP-R1	CTTTCCAGCAGGAGCCGTAT
IP-F2	GCTTGGACTCGTGCCTGGA
IP-R2	AATTGCTACTCCCCGAGCTGTT
*β-actin*-F	CAGTCGGTTGGATGGAGCAT
*β-actin*-R	AGGCAGGGACTTCCTGTAAC
siRNA-Meq^5^	Sense: GCAGACGGACUAUGUAGACTT
	Antisense: GUCUACAUAGUCCGUCUGCTT
siRNA-NC	Sense: UUCUCCGAACGUGUCACGUTT
	Atisense: ACGUGACACGUUCGGAGAATT

Note: ^1^QP denotes primers designed for RT-qPCR; ^2^PRO denotes primers designed for vector construction used for luciferase reporter assay; ^3^CDS denotes primers designed for *Meq* coding sequence used for pcDNA3.1-Meq vector construction; ^4^IP denotes primers designed to conform to the direct binding of *Meq* to *CD30* promoter for ChIP-qPCR; ^5^siRNA-Meq sequences were designed and used as previously described by Levy et al. (2005).

### Antibodies and reagents

DDDDK-Tag rabbit mAb (1:5000) and rabbit anti-β-actin (1:50000) primary antibodies were purchased from ABclonal (Wuhan, China), and HRP-conjugated goat anti-rabbit IgG(*H* + *L*) was purchased from Proteintech (1:5000, IL, USA). Lipofectamine® 3000 transfection kit was purchased from Invitrogen (MA, USA). siRNA-mate plus transfection kit was purchased from Genepharma Biotechnology Co., Ltd. (Suzhou, China). Promega Dual-Glo® Luciferase Assay System was purchased from Promega (MI, USA). SimpleChip® Enzymatic Chromatin IP Kit was purchased from Cell Signaling Technology (MA, USA).

### siRNA synthesis, transfection, and interference efficiency assay

siRNA sequences referred to that by Levy et al. (2005), and were synthesized by Genepharma Biotechnology Co., Ltd. (Suzhou, China). RNA oligo sequence information is shown in Table 1. MSB-1 cells were seeded in 12-well plates. Transfection was performed when cells reached 80 % confluence, following the protocol of the siRNA-mate plus transfection kit manual. At 48 h post-transfection, cell samples were collected, RNA was extracted, and mRNA levels were assessed by RT-qPCR.

### Luciferase reporter assay

The *CD30* promoter region was amplified by PCR using KOD One™ PCR Master Mix (Toyobo, China) and inserted into the pGL4.10 vector using restriction enzyme digestion (HindIII) and ligation. Specifically, nucleotide positions are numbered relative to the transcription start site (TSS) of *CD30*, with the first transcribed base designated as +1, and the genomic sequence spanning approximately 2,000 bp from upstream of TSS to partial exon1 (+352) of *CD30* was defined as the putative promoter region, and the region was amplified by four pairs of primers obtaining four amplicons: CD30 pro (−2054∼+352), truncated region Mut1 (−250∼+352), Mut2 (−950∼+352), and Mut3 (−1450∼+352). The four amplicon sequences were ligand into pGL4.10 to construct four plasmids, pGL4.10-CD30 and pGL4.10-Mut1/2/3. The coding sequence of *Meq* was amplified by PCR and inserted into pcDNA3.1 to construct pcDNA3.1-Meq. All vectors including insertion sequences were verified by Sanger sequencing. All plasmids were extracted by endotoxin-free plasmid extraction kit (Tiangen, China). The DF-1 cells were seeded into 12-well plate at a density of 3 × 10^5^ cells per well. DF-1 cells were transiently transfected with 500 ng pGL4.10-CD30 or pGL4.10-Mut1/2/3, 500 ng pcDNA3.1-Meq, 100 ng pRL-TK using Lipofectamine 3000 (Invitrogen). After 48 h, luciferase activity was measured using a luciferase reporter assay system (Promega), following the manufacturer’s instructions.

### ChIP-qPCR

The *Meq* coding sequence with a 3×Flag epitope tag at the N-terminus was synthesized and cloned into the HindIII site of the pcDNA3.1(+) expression vector to form pcDNA3.1-Meq-3×Flag vector by Tsingke Biotechnology Co., Ltd. (Guangzhou, China). The positive insertion in the pcDNA3.1-Meq-3×Flag vector was confirmed by restriction enzyme digestion and Sanger sequencing. Endotoxin-free pcDNA3.1-Meq-3×Flag vector was extracted by endotoxin-free plasmid extraction kit (Tiangen, Beijing, China) and stored at −20°C for subsequent experiments.

DF-1 cells were seeded at 3 × 10⁵ cells per well in 12-well plates and transfected with 1 μg of pcDNA3.1-Meq-3×Flag plasmid using Lipofectamine 3000 reagent (Invitrogen) per the manufacturer's instructions. Expression efficiency was verified by western blotting at 72 h post-transfection in DF1, and then pcDNA3.1-Meq-3×Flag was transfected into MSB-1 cells by nucleofection. Briefly, 1 × 10⁷ MSB-1 cells were resuspended in 100 μL of Cell Line Nucleofector Solution C (Lonza, Basel, Switzerland) containing 10 μg of the pcDNA3.1-Meq-3×Flag plasmid and electroporated using the Amaxa Nucleofector system (Lonza). Nucleofected cells were immediately transferred to 10-cm culture dishes containing pre-warmed complete medium. At 72 h post-nucleofection, the cells were harvested and processed for chromatin immunoprecipitation using the SimpleChIP® Enzymatic Chromatin IP Kit according to the manufacturer's instructions. Immunoprecipitated DNA was analyzed by qPCR using primers listed in Table 1.

### RNA isolation and cDNA synthesis

Total RNA was isolated from cells using Nuleozol reagent (Macherey-Nagel, Düren, Germany) according to the manufacturer's instructions. First-strand cDNA was synthesized from the extracted RNA using the FastKing gDNA Dispelling RT SuperMix (Tiangen) following the manufacturer's protocol. The synthesized cDNA was stored at −20°C for subsequent analysis.

### Western blotting

Total proteins were extracted from DF-1 cells using SDS-PAGE Protein Loading Buffer (Beyotime, Shanghai, China). Protein samples were separated by SDS-PAGE and electrophoretically transferred onto polyvinylidene difluoride (PVDF) membranes (Millipore, MA, USA). The membranes were blocked and then incubated overnight at 4°C with primary antibodies. Following washing, membranes were incubated with appropriate HRP-conjugated secondary antibodies. Protein bands were visualized using an infrared imaging system and quantified with Image Studio Software.

### Statistical analysis

Statistical analysis of biological replicates was performed by Student’s t-test. Data are presented as mean ± SEM. Statistical significance was set at *p* < 0.05 (*), *p* < 0.01 (**), and *p* < 0.001 (***).

## Results and discussion

### CD30 and Meq were all significantly upregulated in spleens of MDV-infected chickens

**Fig. 1:**
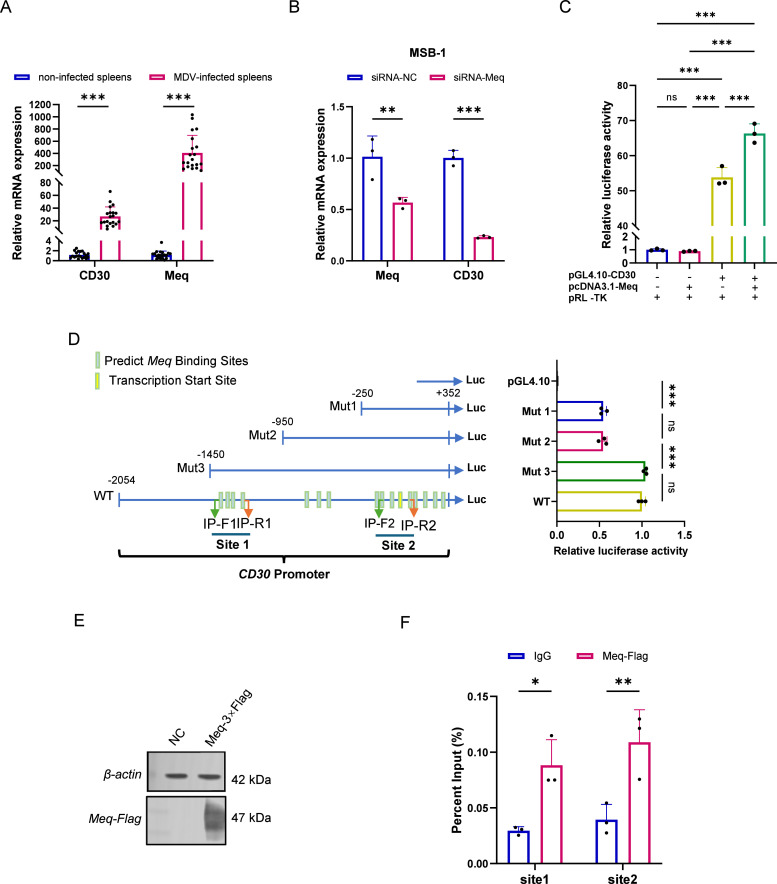
The oncoprotein *Meq* directly bound to the *CD30* promoter and activated its transcription. (A) *CD30* and *Meq* mRNA levels were significantly higher in spleens from MDV-infected chickens with lymphoma compared to those from uninfected controls (*N* = 20), measured by RT-qPCR. (B) Relative mRNA expression of *CD30* in MSB-1 cells with *Meq* knockdown, measured by RT-qPCR. The relative expression levels of target genes were calculated using the 2^(-ΔΔCt) method. Data were normalized to the control group (siRNA-NC). (C) Luciferase reporter assay demonstrating the effect of *Meq* on *CD30* promoter activity. DF-1 cells were co-transfected with a firefly luciferase reporter plasmid driven by the *CD30* promoter (pGL4.10-CD30) and either the *Meq* expression plasmid or an empty vector control. The x-axis of each experimental group represented the types of plasmids transfected. "+" indicated the plasmid containing the target gene, and "−" indicated the transfection of the empty plasmid. (D) Analysis of *Meq*-responsive elements within the *CD30* promoter. The left panel provides a schematic of the *CD30* promoter region, indicating the transcription start site (TSS, yellow box) and putative *AP-1* transcription factor binding sites (Predicted by Burgess et al.(2004), green boxes). The right panel showed the results of a luciferase assay using a series of truncated *CD30* promoter constructs co-transfected with the *Meq* expression plasmid, pinpointing the specific region required for *Meq*-mediated activation. (E) Western blot analysis confirming the expression of 3×Flag-tagged Meq (Meq-3×Flag) in DF-1 cells. A clear band at the expected molecular weight (47 kDa) is visible. (F) ChIP-qPCR was used to detect the binding of *Meq* to the *CD30* promoter in MSB-1 cells. IgG was applied as a negative control. Statistical significance was determined using a Student's t-test. Asterisks denote statistical significance (*p < 0.05, **p < 0.01, ***p < 0.001).

Total RNA was extracted from spleen tissues of SPF chickens without MDV infection (*N* = 20) and from spleen tissues of MDV-infected SPF chickens (*N* = 20). Quantitative expression analysis revealed that *CD30* and *Meq* mRNA levels were significantly higher in spleens from MDV-infected chickens compared to those from uninfected controls (*p* < 0.001, Fig. 1A).

### Knockdown of the MDV oncoprotein Meq downregulated CD30 expression in MSB-1 cells

To investigate the potential regulatory relationship between *Meq* and the host gene *CD30*, we performed a transient knockdown of the *Meq* gene in the MDV-transformed T-cell line MSB-1, which endogenously expresses *Meq*. MSB-1 cells were transfected with siRNAs (siRNA-Meq) specifically targeting Meq with a non-targeting negative control siRNA (siRNA-NC) as a control. At 48 hours post-transfection, the knockdown efficiency was assessed by RT-qPCR. The results showed significant reduction of *Meq* mRNA levels in the siRNA-Meq group, with expression being knocked down by approximately 43.4 % compared to the siRNA-NC group (*p* < 0.05, Fig. 1B). The knockdown of *Meq* led to a significant downregulation of *CD30* mRNA expression (*p* < 0.001, Fig. 1B). These findings indicated that the expression of the viral oncoprotein *Meq* could influence the transcriptional level of the *CD30* gene in MSB-1 cells.

### Meq bound to two regions within the CD30 promoter and activated its transcription

Burgess et al. (2004) predicted the presence of 15 high-stringency *AP-1* transcription factor binding sites, a known interaction partner of *Meq*, in the putative *CD30* promoter, and demonstrated that *Meq* could regulate the expression of *CD30*. However, the precise regions to which *Meq* bound remain undefined. Therefore, we further investigated the *Meq* binding region in *CD30* promoter.

We employed a dual-luciferase reporter assay to test the binding of *Meq* and *CD30* promoter. A reporter plasmid containing the full-length *CD30* promoter sequence (pGL4.10-CD30) was co-transfected into DF-1 cells with either an *Meq* expression vector (pcDNA3.1-Meq) or an empty vector control. The results showed a significant increase in luciferase activity in *Meq* overexpression group compared to the control group, confirming that the Meq protein could robustly activate the *CD30* promoter *in vitro* (Fig. 1C).

To identify the binding regions within *CD30* promoter, we amplified three truncated *CD30* promoter sequences and cloned them into pGL4.10 and constructed three truncated reporter plasmids: pGL4.10-Mut1 (−250 to +352), pGL4.10-Mut2 (−950 to +352), and pGL4.10-Mut3 (−1450 to +352). Each of pGL4.10-Mut1/2/3 or an empty vector control (pGL4.10) was co-transfected with the pcDNA3.1-Meq plasmid into DF-1 cells.

Luciferase activity assays revealed that pGL4.10-Mut1 showed a significantly high luciferase activity compared with the empty vector, and pGL4.10-Mut3 showed a significant increase compared to the empty vector and pGL4.10-Mut2 (*p* < 0.001). This suggests the regulatory region binding with *Meq* was located in −1450 ∼ −950, and −250 ∼ +352 within the *CD30* promoter (Fig. 1D).

To determine if *Meq* directly bound to these identified regulatory regions *in vitro*, we performed the ChIP-qPCR assay. A 3×Flag-tagged Meq expression vector (pcDNA3.1-Meq-3×Flag) was transfected into MSB-1 cells. Chromatin was then immunoprecipitated using an anti-Flag antibody or IgG antibody, which acted as a control. Two pairs of specific primers were designed to amplify two regions from the ChIP samples. The results showed a significant enrichment of *Meq* binding at two regions (*p* < 0.01) in anti-Flag antibody group compared with IgG group (Fig. 1E, F). These findings confirmed that *Meq* directly bound to the *CD30* promoter at the regions (−97 ∼ +75 and −1383 ∼ −1162) to drive its expression.

In this study, we found that *Meq* acted as a transcriptional regulator capable of modulating *CD30* expression *in vitro* experiments. This observation aligns with earlier research, which reported that *Meq* acted as a bZIP transcription factor that forms dimers with cellular partners such as *c-Jun* and *c-Fos* to regulate gene transcription (Levy et al., 2003). Structural features of *Meq* suggested potential binding to AP-1-like consensus sequences within the *CD30* promoter region (Burgess et al., 2004). These previous studies were consistent with our results that positive correlation between *Meq* expression and *CD30* upregulation were observed in MDV-transformed lymphocytes.

In our previous study, we found that *CD30* was strongly upregulated in MD lymphomas, with hypomethylation of the *CD30* promoter in infected chicken compared with non-infected controls (Li et al., 2015; Zhang et al., 2012). Generally, low methylation in the promoter region facilitates the binding of transcription factors (Jones and Laird, 1999), so we speculated that hypomethylation of *CD30* promoter might facilitate *Meq/c-Jun* heterod binding and further amplify *CD30* activation, enhancing oncogenic signaling.

The upregulation of *CD30* in MDV-infected cells is of particular interest, as *CD30* is a well-established tumor marker in both avian and human cancers. In humans, *CD30* is highly expressed in various lymphoma subtypes, particularly in Hodgkin's lymphoma and anaplastic large-cell lymphoma (ALCL). *CD30* serves as a critical cell surface marker for these lymphomas and has been linked to poor prognosis in certain types of lymphoid malignancies. Importantly, *CD30*'s role in tumorigenesis extends beyond its expression as a mere biomarker; it is involved in key signaling pathways, including the activation of *NF-κB* and *JAK/STAT*, which regulate cell proliferation, survival, and immune responses (Nakashima and Uchimaru, 2023).

Recent advancements have shown that *CD30*-targeting therapies, and brentuximab vedotin, an antibody-drug conjugate (ADC), have significantly improved treatment outcomes for *CD30*-positive lymphomas (Jiang et al., 2025). Given the strong upregulation of *CD30* in MDV-induced lymphomas, it is plausible that *CD30*-targeted therapies could be explored as potential options for controlling MD.

In conclusion, this study elucidates the transcriptional regulatory relationship between the MDV oncoprotein *Meq* and the chicken gene *CD30*. We verified the direct binding of *Meq* to the *CD30* promoter and identified the binding regions through luciferase reporter assays and ChIP-qPCR, confirming *Meq* as a direct transcriptional activator of *CD30*. These findings provide a foundation for further functional analyses of host factors involved in MDV pathogenesis and suggest that *CD30* may serve as a potential therapeutic target in MDV-associated lymphomas..

## Disclosures

The authors declare that they have no conflict of interest.

## CRediT authorship contribution statement

**Yushuang Du:** Writing – review & editing, Writing – original draft, Validation, Methodology, Investigation, Formal analysis, Conceptualization. **Gang Zheng:** Writing – review & editing, Methodology. **Yuqin Yang:** Writing – review & editing. **Jiannan Zhang:** Writing – review & editing, Methodology. **Juan Li:** Writing – review & editing. **Ling Lian:** Writing – review & editing, Resources, Project administration, Funding acquisition, Conceptualization.
